# A Case Report of a National Judo Champion: Is Return to High-Level Contact Sports Possible After Meniscus Allograft Transplantation?

**DOI:** 10.7759/cureus.56764

**Published:** 2024-03-23

**Authors:** Fiona Vanbiesbroeck, Jasper Vandenrijt, Francis Van Glabbeek, Peter Verdonk, Christiaan H Heusdens

**Affiliations:** 1 Orthopaedics, University of Antwerp, Antwerp, BEL; 2 Orthopaedics, Antwerp University Hospital, Antwerp, BEL; 3 Orthopaedics, AZ (Algemeen Ziekenhuis) Monica, Antwerp, BEL

**Keywords:** case report, high-level, judo, contact sports, meniscus allograft transplantation

## Abstract

Meniscus allograft transplantation (MAT) is a surgical procedure reserved for (relatively) younger individuals who remain symptomatic after the resection of a voluminous part of the meniscus. Return to sports and certainly the level of sport post-MAT are highly variable. We present a unique case of a national judo champion who was able to compete at the highest level following MAT. Considerations regarding the rehabilitation and follow-up of this patient are provided, and the risk of rerupture is discussed. Although returning to high-level contact sports post-MAT is possible, the risk of rerupture should be considered.

## Introduction

Meniscal allograft transplantation (MAT), initially conducted in 1984, serves as a possible therapeutic choice for younger patients (approximately < 50 years old) afflicted with isolated unilateral femorotibial knee pain confined to the medial or lateral compartment following a past (sub)total or functionally comparable meniscectomy [1,2]. Additionally, the absence of advanced joint arthritis or osteoarthritis exhibiting degenerative alterations, malalignment, knee instability, systemic or localized infection, synovial disorders, autoimmune diseases or inflammatory arthritis, bipolar cartilaginous lesions, knee arthrofibrosis, or skeletal immaturity is imperative. Preferably, individuals are non-smokers and maintain a BMI below 30 [1-5]. Graft preservation options include (i) lyophilization (freeze-drying), often in combination with irradiation; (ii) freezing (deep-frozen or fresh-frozen); or (iii) cryopreservation [1,2,5]. Both graft survival rates and rates of return to sporting activities demonstrate considerable variability, with graft survival rates ranging from 45% to 73.5% at 10 years and a return to sport percentage ranging from 77% to 100% [1,6-10]. Meniscal scaffolds could offer an alternative for younger symptomatic individuals with a partial meniscal defect and an intact peripheral rim; nevertheless, randomized controlled trials assessing long-term outcomes are currently insufficient [11]. This study presents a case of a national judo champion who underwent MAT surgery and successfully resumed competition at the equivalent pre-injury level. The patient's medical history leading up to MAT surgery, subsequent rehabilitation, resumption of sporting activities, and post-treatment follow-ups will be outlined. This case report aims to provide physicians with insights regarding the risks and benefits associated with returning to high-level contact sports following MAT intervention.

## Case presentation

**Figure 1:**
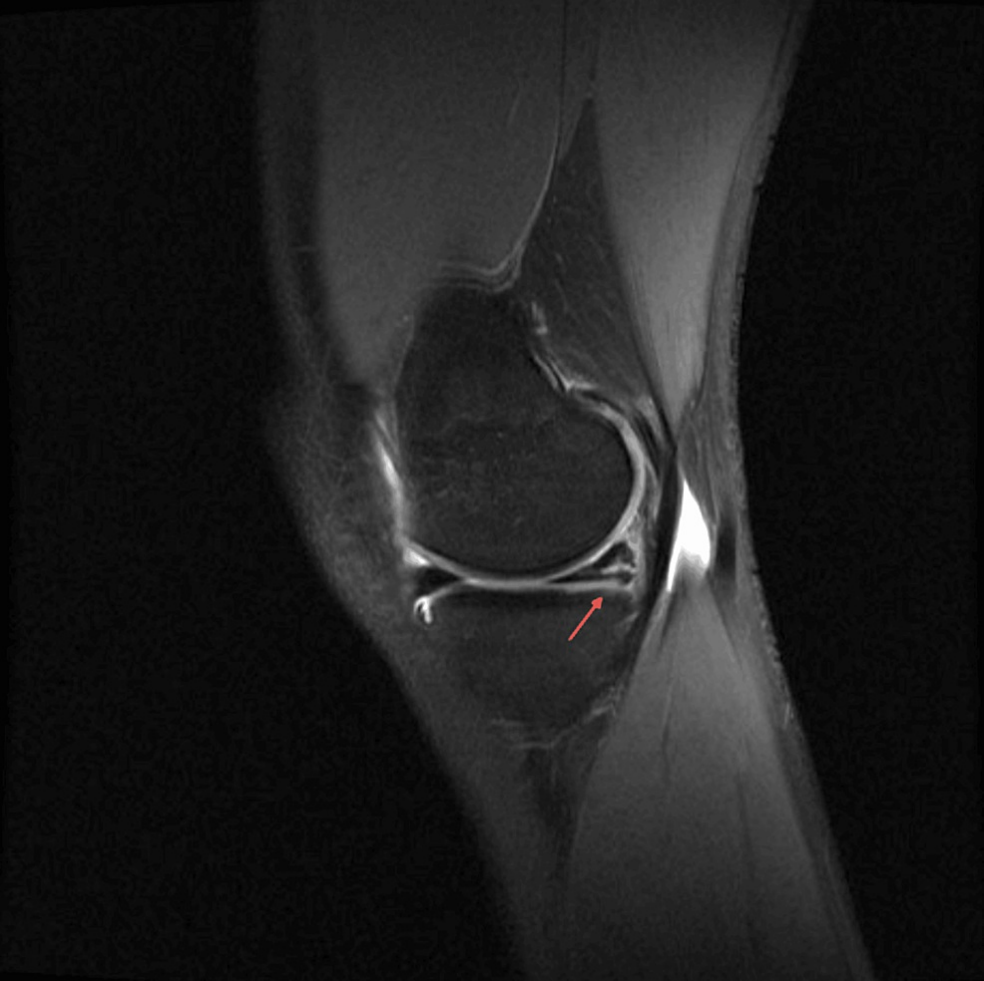
Initial MRI Horizontal tear (red arrow) at the posterior horn of the medial meniscus. Sagittal view of the left knee. MRI: Magnetic resonance imaging

We present a unique case of a 16-year-old female national judo champion who underwent MAT at our institution. Her medical history involved two previous surgeries: a meniscus suturing procedure and a partial medial meniscectomy. Initially, the patient sustained a meniscal rupture during judo training while executing the Ippon Seoi Nage Drop technique. This technique required her to rotate her left knee while falling on both knees with the weight of a training partner on her back. The initial treatment involved suturing the horizontal tear in the posterior horn of the medial meniscus three months subsequent to the incident (Figure 1).

**Figure 2:**
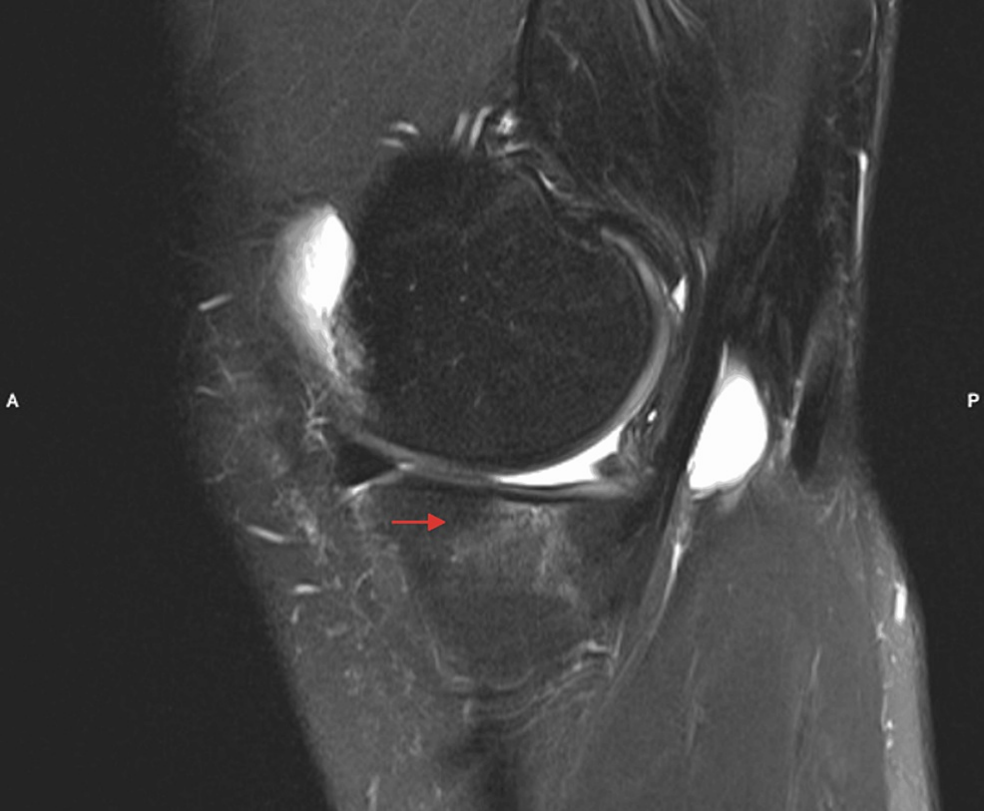
Pre-MAT MRI Status post partial medial meniscectomy. Bone marrow edema (red arrow) and increased signal intensity in the remaining meniscus. Sagittal view of the left knee, medial meniscus. MAT: Meniscus allograft transplantation; MRI: magnetic resonance imaging

The patient experienced a failure of the sutured meniscus, leading to a partial medial meniscectomy one year after the initial meniscus suturing (Figure 2). Subsequent to the meniscectomy, the patient continued to experience persistent pain, prompting her to seek a second opinion one and a half years after the meniscus tear. At the time of the consultation, the patient was a multiple national champion with international victories. Due to her young age and the presence of postmeniscectomy syndrome, she was placed on the transplant list for medial MAT. Seven months later, in April 2018, the medial MAT procedure was performed.

Meniscus allograft transplantation

**Figure 3:**
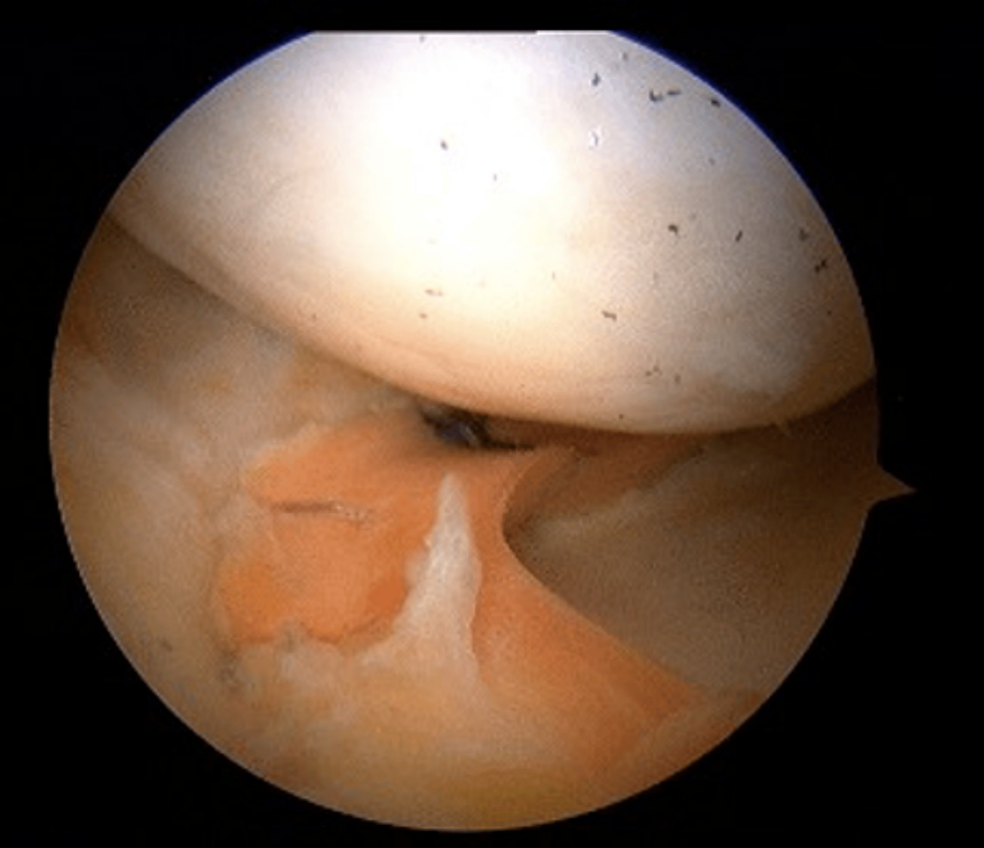
MAT surgery Arthroscopic view of the fixed graft. Anterior view of the left knee, medial meniscus. MAT: Meniscus allograft transplantation

The medial meniscus allograft transplantation (MAT) was conducted using a combination of open and arthroscopic techniques, as outlined by Spalding et al. [12]. A non-irradiated, fresh-frozen allograft (donor meniscus of human origin) was utilized (Figure 3). Following the procedure, platelet-rich plasma (PRP) was injected into the joint to facilitate the healing process.

Follow-up

The patient's mobility was limited by an unloader brace, and weight-bearing was not allowed for six weeks. The unloader brace was required to be worn continuously for the initial six weeks; subsequently, it was only necessary to wear the brace during the day for up to six months following the operation, with no restrictions in range of motion. A rigorous rehabilitation protocol was adhered to, with a gradual introduction of exercises that demanded increased strength and stability. Table 1 provides a summary of when the rehabilitation goals or exercises were achieved or introduced postoperatively.

**Table 1:** Rehabilitation goals and exercises post-MAT MAT: Meniscus allograft transplantation

Rehabilitation goal/exercise	Time post-MAT
90° knee flexion	Week 8
Cycling	Week 12
Proprioception exercises	Month 2
Full knee flexion	Month 3
Jumping exercises	Month 4
Running	Month 5
Judo training (technique)	Month 10
Judo training (technique and fighting)	Month 11

**Figure 4:**
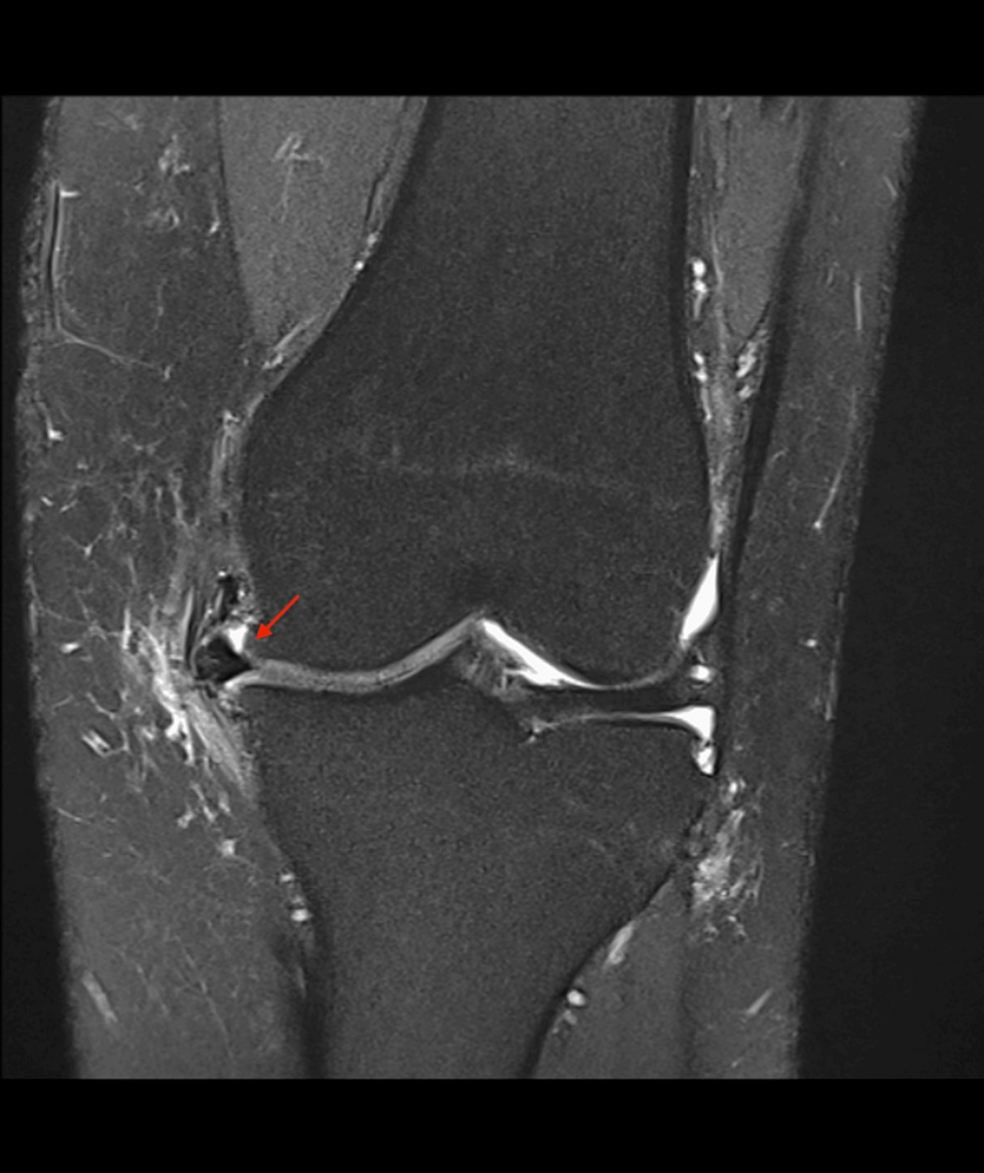
MRI three months post-MAT A 3 mm radial displacement (red arrow) of the meniscus corpus is visible. Coronal view of the left knee, medial meniscus. MAT: Meniscus allograft transplantation; MRI: magnetic resonance imaging

In the rehabilitation phase, a follow-up magnetic resonance imaging (MRI) was carried out three months after MAT, revealing a radial displacement of the meniscus corpus measuring 3 mm; however, no clinical symptoms or implications were observed (Figure 4). At the 10-month post-MAT mark, as the patient resumed judo training, no issues were reported. Rotations, deep flexion, and pivoting maneuvers did not cause pain, instability, or discomfort. The patient only experienced difficulty with barbell squats during power training, which was consequently avoided, although it did not impact any judo techniques.

One year after MAT, the patient reported discomfort in the medial aspect of the knee when pressure was applied to the medial collateral ligament (MCL) area. The MRI results at this stage were similar to those from three months post-MAT. Due to this heightened sensitivity, the inside-out sutures were removed via the medial incision, and knee arthroscopy was performed simultaneously to assess the graft, which was found to be fully healed. Following a standard rehabilitation protocol, the patient returned to competition at 17 months post-MAT, securing the national champion title for the sixth time and achieving international victories.

At two and a half years post-MAT, isokinetic tests using an isokinetic dynamometer were conducted for injury prevention purposes. These tests still demonstrated a 26% difference in quadriceps strength between the two lower limbs, with the operated leg at a disadvantage, even after completing the MAT rehabilitation program (Table 2).

**Table 2:** Isokinetic test results two and a half years post-MAT Isokinetic test: dynamic muscle strength test that measures forces at a constant speed of movement; concentric: muscle contraction leading to shortening of the muscle; eccentric: muscle contraction leading lengthening of the muscle; F_max_: maximum force; N.m: newton-meter; H/Q: hamstring/quadriceps; H_ecc_/Q_conc_: hamstring eccentric /quadriceps concentric; °/s: degrees per second; MAT: meniscus allograft transplantation

Isokinetic test results			
Quadriceps F_max_ (N.m)			
	Right	Left	Difference (%)
Concentric 60°/s	148	122	- 26%
Concentric 240°/s	85	80	- 5%
Hamstrings F_max_ (N.m)			
Concentric 60°/s	65	66	+ 1%
Concentric 240°/s	39	38	- 1%
Eccentric 30°/s	79	85	+ 6%
Agonist/antagonist ratio (H/Q-ratio)			
Concentric 60°/s	0.44	0.54	/
Concentric 240°/s	0.46	0.47	/
H_ecc_/Q_conc_	0.93	1.06	/

**Figure 5:**
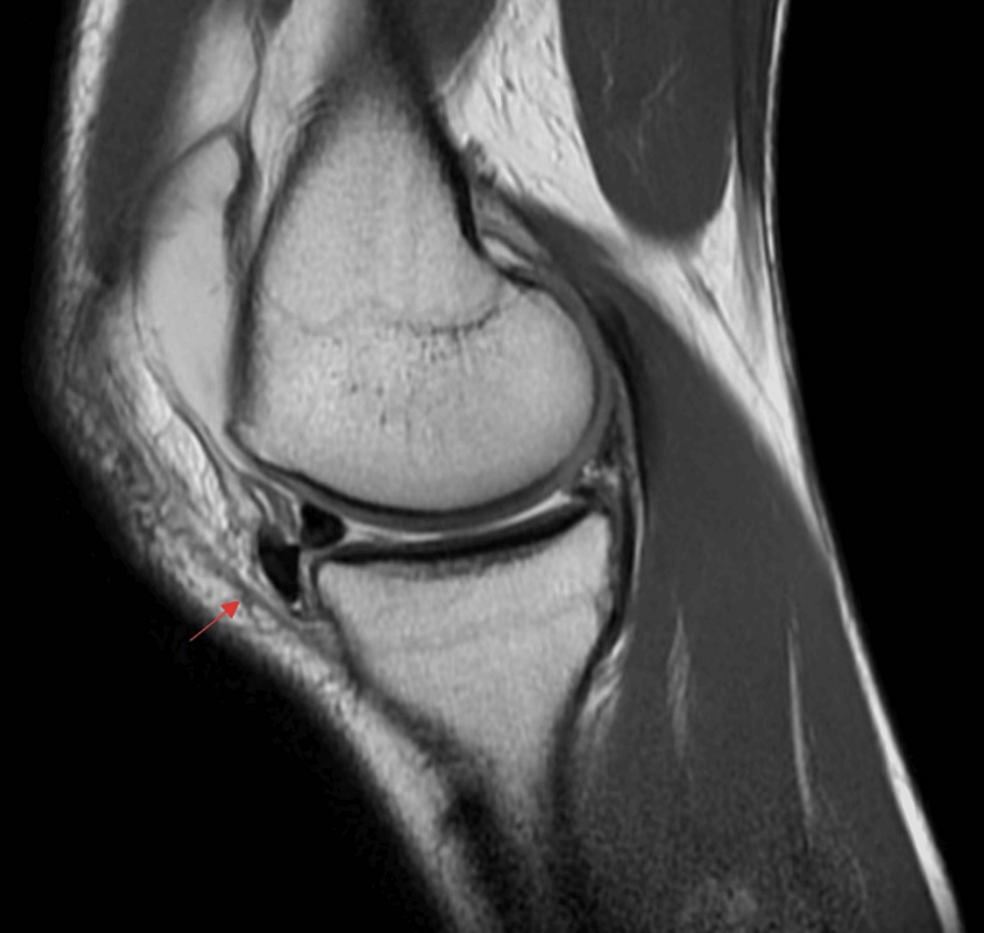
Graft tearing Magnetic resonance imaging showing anterior luxation of the graft (red arrow) resulting in a locked knee. Sagittal view of the left knee, medial meniscus.

Four years and three months after MAT, during judo practice, the patient experienced a subluxating sensation of the medial meniscus out of the knee joint followed by an immediate reduction to its normal position. The patient completed the training session without encountering further issues. The next day, minor swelling occurred, which resolved spontaneously. Two days later, she participated in a major competition without any hindrance from the incident. However, the following week, while rotating on the left leg, she felt a tearing sensation in the knee. This immediately led to significant swelling, with the knee joint remaining entirely locked at a 45° flexion angle. An MRI revealed an anterior luxated bucket handle tear (Figure 5). As there was no spontaneous reduction of the meniscus, knee arthroscopy was conducted two days later.

**Figure 6:**
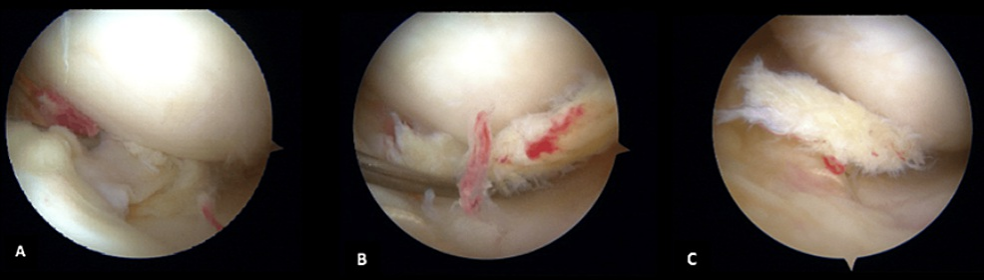
Intraoperative findings of torn meniscus allograft Intraoperative view of the torn graft with degenerative fraying. Articular cartilage showed no lesions. Anterior view of the medial meniscus of the left knee.

In addition to the anterior luxated bucket handle tear, a flap tear of the mid-portion was observed, as well as degenerative fraying and horizontal tearing of the posterior horn of the meniscus (Figures 6A-6C). Initially, a meniscectomy of the mid-portion was conducted; however, due to instability, the remaining posterior and mid-portions were also resected. A stable part of the anterior horn was retained in place.

**Figure 7:**
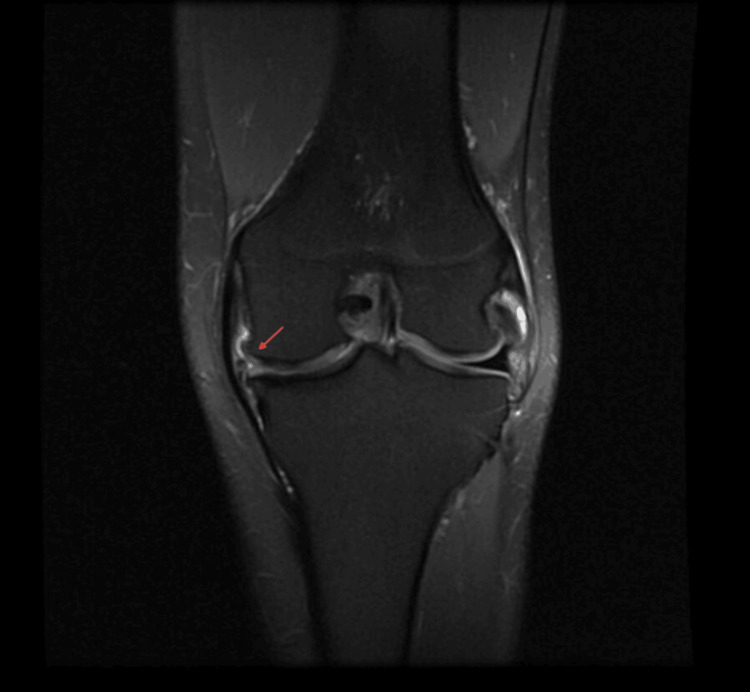
Magnetic resonance imaging status post graft resection Subtotal meniscectomy of the medial meniscus (red arrow). Coronal view of the left knee.

The patient adhered to a standard postmeniscectomy rehabilitation protocol. However, following the meniscectomy, she continued to experience sharp pain in the posterolateral corner of the knee joint during knee flexion. Additionally, prolonged standing was painful (Figure 7). Subsequently, the patient decided to conclude her career as a high-level athlete and expressed willingness to undergo a second MAT. Despite the rerupture, she expressed high satisfaction with the outcomes of the initial MAT, which effectively alleviated the pain and enabled her to resume high-level judo for nearly four years following rehabilitation. Recently, the patient underwent a second MAT. Rehabilitation progressed smoothly, and there has been an improvement in knee pain during daily activities.

## Discussion

Returning to high-level contact sports such as judo post-MAT is an important consideration. The literature on returning to sports subsequent to MAT remains a controversial topic. Grassi et al. reported a 77% return to sports rate, with a 67% return to the preinjury level [8]. Hurley et al. reported similar percentages [9]. Additionally, Bonanzinga et al. documented a 100% return to sport and a 69% return to the preinjury level in professional athletes [10]. However, most reports regarding return to sports have been for activities such as running, soccer, basketball, and cycling, with no documented cases of return to judo at a high level [8,10,13]. The nature of judo, characterized by constant rotational movements and significant knee joint stress, particularly due to falling and carrying a training partner, makes the knee joint the most common site of injury in judo athletes [14]. Reported MAT graft survival rates vary widely, with rates ranging from 45% [7] to 73.5% [6] at 10 years and 19% [7] to 60.3% [6] at 15 years of follow-up. In a study by Bin et al., distinction between medial and lateral MAT revealed graft survival rates of 85.8% and 89.5% for medial and lateral MAT, respectively, at midterm follow-up (5-10 years), and rates of 52.6% and 56.6% for medial and lateral MAT, respectively, at long-term follow-up (>10 years) [15]. The influence of the athlete's performance level and sport-specific loading on graft survival post-MAT has not been thoroughly researched. Investigating the impact of returning to sport as a prognostic factor for graft survival, particularly distinguishing between contact and non-contact sports, is an avenue for further research. It is crucial to weigh the promising prospect of returning to sports against the risk of rerupture, in particular when partaking in a high-level contact sport such as judo. Open communication between the surgeon and the athlete regarding the potential risks (such as retearing the meniscus and lengthy rehabilitation) and benefits (such as pain resolution and resuming sports) post-MAT is essential. Unfortunately, the literature still lacks graft survival rate data pertaining to highly athletic populations.

Even at two and a half years post-MAT, a 26% difference in quadriceps strength persisted. It is unclear whether this imbalance, to the detriment of the operated leg, contributed to the early graft failure. McLeod et al. documented quadriceps weakness for up to four years following meniscectomy [16]. Consequently, even after completing a comprehensive rehabilitation program, ongoing deficits require close monitoring, especially for individuals intent on returning to sports activities. The hamstring/quadriceps ratio can serve as a valuable tool for screening hamstring injuries [17]. Ha et al. utilized isokinetic muscle strength, among other parameters, to assess patient satisfaction post-MAT, finding a significant association between an isokinetic 60°/s extension deficit and patient satisfaction [18]. Additionally, Darbandi et al. demonstrated the utility of isokinetic dynamic tests in predicting lower limb injuries in elite judo athletes [19]. Although post-MAT isokinetic dynamic tests have been used to assess patient satisfaction, their prognostic value in terms of graft survival has not yet been explored. The role of isokinetic tests in the post-MAT follow-up represents an interesting subject for further research.

The two primary types of allograft fixation are soft tissue or bony fixation using either bony plugs or a bony bridge. The bony fixation group may offer biomechanical superiority and a lower complication rate. However, there is currently no evidence demonstrating superiority in terms of clinical outcomes for patients. Hence, the International Meniscus Reconstruction Experts Forum (IMFREF) has concluded that there is no superiority between the two techniques [20].

## Conclusions

This is the first case report on a patient returning to high-level contact sport post-MAT. After an extensive rehabilitation program, the patient was able to return to compete at the same pre-injury level and even became a national judo champion. However, a re-arthroscopy had to be performed one year post-MAT to remove the inside-out sutures due to discomfort over the MCL area. Furthermore, isokinetic tests demonstrated a 26% deficit in quadriceps strength two and a half years post-MAT. Four years post-MAT, a rerupture occurred while executing a rotational movement in judo practice. The potential for resuming high-level contact sports post-MAT is viable but the associated risk of rerupture and the lengthy rehabilitation period should be carefully considered against the advantages of pain relief and resumption of sporting activities. These considerations should be thoroughly discussed between the patient and the surgeon.
